# Plasma Proteomics-Based Discovery of Mechanistic Biomarkers of Hyperbaric Stress and Pulmonary Oxygen Toxicity

**DOI:** 10.3390/metabo13090970

**Published:** 2023-08-23

**Authors:** Kyle J. Mahoney, Jacob S. Bowie, Austin E. Ford, Neranjan Perera, Yasuki Sekiguchi, David M. Fothergill, Elaine C. Lee

**Affiliations:** 1 Department of Kinesiology, University of Connecticut, Storrs, CT 06269, USA; kyle.j.mahoney@uconn.edu (K.J.M.); jacob.bowie@uconn.edu (J.S.B.); neranjan007@gmail.com (N.P.);; 2 Naval Submarine Medical Research Laboratory, Groton, CT 06349, USA; david.m.fothergill.civ@health.mil

**Keywords:** hyperoxia, oxygen toxicity, proteomics, decompression stress

## Abstract

Our aim was to identify proteins that reflect an acute systemic response to prolonged hyperbaric stress and discover potential biomarker pathways for pulmonary O_2_ toxicity. The study was a double-blind, randomized, crossover design in trained male Navy diver subjects. Each subject completed two dry resting hyperbaric chamber dives separated by a minimum of one week. One dive exposed the subject to 6.5 h of 100% oxygen (O_2_) at 2ATA. The alternate dive exposed the subjects to an enhanced air nitrox mixture (EAN) containing 30.6% O_2_ at the same depth for the same duration. Venous blood samples collected before (PRE) and after (POST) each dive were prepared and submitted to LC-MS/MS analysis (2 h runs). A total of 346 total proteins were detected and analyzed. A total of 12 proteins were significantly increased at EANPOST (vs. EANPRE), including proteins in hemostasis and immune signaling and activation. Significantly increased proteins at O_2_PRE (vs. O_2_POST) included neural cell adhesion molecule 1, glycoprotein Ib, catalase, hemoglobin subunit beta, fibulin-like proteins, and complement proteins. EANPOST and O_2_POST differed in biomarkers related to coagulation, immune signaling and activation, and metabolism. Of particular interest is (EANPOST vs. O_2_POST), which is protective against oxidative stress.

## 1. Introduction

Hyperbaric oxygen (HBO) exposure during clinical treatment or during diving activities results in reactive oxygen (ROS) and reactive nitrogen (RNS) species that drive the cellular signaling, cytoprotective, and antioxidant responses during exposure. The positive or negative nature of the oxidative stress that distinguishes therapeutic or ergogenic use versus susceptibility to injury or stress-induced damage depends on the partial pressure of oxygen breathed and the duration of exposure (i.e., the oxygen dose) as well as individual susceptibility to oxygen toxicity.

Although hyperbaric oxygen is currently an approved treatment for 14 medical conditions [1], only a few studies have assessed the effects of hyperbaria and hyperoxic stress at the gene expression level. Repetitive HBO exposure in mice affects biological processes in the lungs such as response to wounding, extracellular matrix, vasculature development, and immune response [2]. Target prediction of differentially expressed genes in another murine study suggested that regulation of gene expressions of dopamine metabolism and nitric oxide synthesis were significantly affected by carbon monoxide poisoning and HBO treatment [3]. HBO exposure to in vitro HIV-1- infected cells led to several signaling processes in the cell, such as those stimulating NFκb, interferon α, and p21, all of which affect each other to inhibit HIV virus replication [4]. Human proteomic or omics studies related to prolonged hyperbaric oxygen exposure and pulmonary oxygen toxicity in humans do not exist in the current literature. Thus, the aim of our study was to characterize the plasma proteomic response to prolonged hyperbaric, hyperoxic stress, and to discover potential biomarkers of pulmonary O_2_ toxicity. 

## 2. Materials and Methods

The study was a double-blind, randomized, and sham-controlled crossover design in 14 male US Navy-trained diver subjects. The mean ± SD age and weight were 33.1 ± 7.5 years and 84.3 ± 11.8 kg, respectively. Each subject completed two dry resting dives within the hyperbaric chamber located at the Pressurized Submarine Escape Trainer, Naval Submarine School, Groton, CT. One dive exposed the subjects for 6.5 h to 100% O_2_ at 2 ATA (HBO2). Based upon pulmonary oxygen toxicity model predictions of decrements in the vital capacity [5] and previous long-duration dives conducted at the Naval Submarine Medical Research Laboratory, this dive profile was expected to elicit a mild but reversible level of pulmonary oxygen toxicity in at least half of the subjects while at the same time keeping the risk of a seizure from central nervous system oxygen toxicity to a minimum.

The control or sham comparison dive was an enhanced air nitrox (EAN) dive in which the subjects breathed a 30.6% O_2_ balance nitrogen mixture at the same depth for the same duration. A 30.6% nitrox mixture rather than compressed air is breathed to maintain a square wave dive profile and avoid staged decompression on either air or oxygen. Avoiding staged decompression phases supported the intent to keep the subjects and investigative team blinded to the two gas conditions. Diving on a 30.6% FiO2 nitrox mixture and decompressing in accordance with the depth time profile of an air decompression table (without recalculating the equivalent air depth) will reduce the decompression stress of the dive and likely result in lower levels of VGE than if the same dive profile had been performed breathing air. However, in our calculation of the No-Decompression limit (in accordance with the U.S. Navy dive manual), we took into account the equivalent air depth based on the nitrogen content of the breathing mixture. During the sham dive in which the subjects will breathe 0.309 ATA 02 nitrox gas mix at 2 ATA, the partial pressure of nitrogen that they will be breathing will be 1.382 ATA which is equivalent to an air depth of 25 fsw. Breathing air at 2 ATA for the same duration is an exceptional exposure dive according to the US. Navy Dive (Rev 7 manual) requires a significant period of decompression at 20 fsw.

The study protocol was reviewed and approved by the Naval Submarine Medical Research Laboratory institutional review board. All subjects were informed of the risks of the study and signed an informed consent. As one of the primary risks of the study was oxygen toxicity, they were briefed on the signs and symptoms of both CNS as well as pulmonary oxygen toxicity and asked to report any onset of symptoms to the inside dive tender. Additionally, each subject was debriefed after each dive by the principal investigator for any signs or symptoms relating to pulmonary oxygen toxicity that they currently had or had experienced during the dive.

Subjects were instructed to avoid drinking alcohol and conduct heavy exercise 24 h before each dive. They were also instructed to avoid any additional hypo or hyperbaric exposures during the study period. During each dive, subjects were allowed 15 min approximately mid-way through the dive to eat a low nitrate-containing lunch. During this lunch period, the subjects remained at depth in the chamber and were allowed to take off their oral nasal mask to breathe chamber air. Thus, the total bottom time for the HBO2 and EAN dive including the air break was 6 h and 45 min. The two dives were conducted at the same time of day (dive start times ranged from 08:18 to 10:10) and were separated by a minimum of one week apart. 

Venous blood samples were drawn from the antecubital veins of the subjects’ arm within 30 min of the start of each dive (PRE) and approximately 30–45 min after the subjects had surfaced from each dive (POST). The blood samples were collected and processed according to manufacturer instructions for the BD^TM^ P100 Blood Collection System (BD Biosciences, Franklin Lakes, NJ, USA). This collection system supports the collection and preservation of plasma protein with filtration to ensure the best sample preservation and storage prior to proteomics assays. Protein extraction and depletion (High Select™ Top14 Abundant Protein Depletion Mini Spin Columns, ThermoFisher Scientific, Inc., Waltham, MA, USA) were conducted prior to trypsin digestion and evaluation by mass spectrometry on Q Exactive HF Mass Spectrometer (2 h per sample, Thermo Scientific, Inc.). MaxQuant was searched with a fragment ion mass tolerance of 20 PPM and a parent ion tolerance of Unknown. Carbamidomethyl (C) of cysteine was specified in MaxQuant as a fixed modification. Gln->pyro-Glu of the n-terminus, deamidation (NQ) of asparagine and glutamine, oxidation (M) of methionine, and acetyl (Protein N-term) of the n-terminus were specified in MaxQuant as variable modifications. Uniprot IDs (MaxQuant: Majority protein IDs) were matched to a list of 346 unfiltered proteins detected (0.0% minimum, 10.8% Decoy False Discovery Rate (FDR) for 381,213 spectra (0.0% minimum, minimum of 2 peptides, 0.0% Decoy FDR) across all samples. Scaffold Q + S (v.5.3.0, proteomesoftware.com (accessed 1 February 2023) was used to quantify proteins. Scaffold was used to conduct ANOVA to compare all conditions in one analysis and comparison of independent conditions by two-tailed paired t-tests (significance at *p* < 0.05 (uncorrected *p*-value)) in average precursor intensity (fold-change relative to control or alternate condition for each comparison). Protein identifications were accepted if they contained at least two identified peptides. Proteins that contained similar peptides and could not be differentiated based on MS/MS analysis alone were grouped to satisfy the principles of parsimony. Proteins sharing significant peptide evidence were grouped into clusters. Scaffold analysis workflow uses a pipeline of several peptide and protein validation methods following an initial database-search engine analysis [6,7,8]. Pathway analysis and functional annotation analyses were conducted using String v11.5 (string-db.org (accessed 1 February 2023).

## 3. Results

While all 14 subjects conducted both dives, delays in the availability of the P100 Blood Collection System prevented blood collection from a few subjects conducting the first few scheduled dives. Hence, PRE and POST dive blood samples were collected for only nine of the subjects for the O_2_ dive and 11 of the subjects for the EAN dive. During the EAN dive, none of the subjects reported any symptoms of pulmonary oxygen toxicity. During the O_2_ dive, one subject reported symptoms of dry cough 3 h into the dive that continued to worsen over the next couple of hours. At 4.5 h into the dive, he reported discomfort in breathing and difficulty taking full inspiration. Following a medical evaluation in the chamber, the subject was decompressed to the surface after a total time of 304 min on O_2_. This was the only subject with severe signs and symptoms of pulmonary oxygen toxicity that resulted in early termination of the O_2_ dive. Three subjects reported moderate symptoms of pulmonary oxygen toxicity following the O_2_ dive that included: “burning irritation in the chest, sensations of the need to cough, feeling out of breath, or the inability to take a deep breath”. Six subjects had relatively mild symptoms of pulmonary oxygen toxicity (e.g., minor discomfort on inhalation) and only four subjects reported that they did not experience any symptoms of pulmonary oxygen toxicity following the O_2_ dive. Pulmonary oxygen toxicity symptoms for the nine subjects for which proteomic blood samples were analyzed pre and post HBO included four with mild symptoms, two with moderate symptoms, one with severe symptoms, and two with no symptoms. 

### 3.1. Pre-Exposure Conditions Included Proteomic Differences in Proteins between EAN and Hyperbaric O_2_ (EANPRE vs. O_2_PRE)

Despite that baseline (PRE) conditions represented a presumably unstressed resting state among participants, five proteins (Table 1) were significantly (*p* < 0.05) higher in O_2_PRE (vs. EANPRE). Network analysis indicates four significant edges (PPI (protein and protein interactions) enrichment *p*-value 2.11 × 10^−6^) with functional enrichments in gene ontology related to chemokine, cytokine, and coagulation function (Table 2) and three reactome pathways (Table 3). 

### 3.2. Proteomic Differences with Hyperbaric O_2_ Exposure (O_2_PRE vs. O_2_POST)

Presence/absence analysis revealed five proteins uniquely present in O_2_PRE and three uniquely present in O_2_POST (but not significantly associated with any networks FDR > 0.05) (Supplemental Table 1). Network analysis of the eight proteins revealed no significant networks and no functional enrichments (PPI enrichment *p*-value = 1.0).

Based on the quantitative profile of the 346 proteins, nine were significantly expressed in O_2_PRE and one was uniquely high in O_2_POST (*p* < 0.05) (Table 4). Network analysis of the nine highly expressed proteins in O_2_PRE indicated two significant edges (PPI enrichment *p*-value 0.186) with functional enrichments in gene ontology related to blood coagulation (Table 5). Reactome pathways significantly (FDR < 0.05) associated with the nine proteins were regulation of the immune system (HSA-168256) and the innate immune system (HSA-168249). Significantly associated KEGG (Kyoto Encyclopedia of Genes and Genomes) pathways (FDR < 0.05) were related to complement and coagulation cascades (hsa04610) and prion disease (hsa05020). The one highly expressed protein in O_2_POST was apolipoprotein D, known for its biological functions associated with lipid metabolism and neuroprotection.

### 3.3. Proteomic Differences between EANPRE vs. EANPOST

Quantitative profile analysis of EANPRE vs. EANPOST revealed four and 12 proteins significantly highly expressed in EANPRE and EANPOST, respectively (Table 6). Network analysis of the four highly expressed proteins in EANPRE revealed no significant networks (PPI enrichment *p*-value = 1.0).

Network analysis of the 12 highly expressed proteins in EANPOST indicates 24 significant edges (PPI enrichment *p*-value < 1.0 × 10^−16^) with many different functional enrichments in gene ontology (Table 7). Reactome pathways significantly (FDR < 0.05) associated with the 12 proteins were hemostasis (HSA-109582), platelet degradation (HSA-114608), intrinsic pathway of fibrin clot formation (HAS-140837), and defective F9 activation (HSA-9673221). The only significantly associated KEGG pathway (FDR < 0.05) was the regulation of complement and coagulation cascades (hsa04610).

### 3.4. Proteomic Differences between Post Conditions (EANPOST vs. O_2_POST)

We conducted an ANOVA analysis to determine proteins that were significantly different when all four conditions (EANPRE, EANPOST, O2PRE, and O2POST) were entered into the model. There were six proteins that were significantly different (*p* < 0.05): (1) the HBB, cluster of hemoglobin subunit beta which contributes to oxygen transport, elevated in (2) AMBP, alpha-1 microglobulin protein, antioxidant, and tissue repair protein with reductase, heme-binding, and radical scavenging activity, APOD, (3) apolipoprotein D, (4) NCHL-1, neural cell adhesion molecule, an uncharacterized protein with putative function or structure similar to NCHL-1, (5) SERPIND1 or HEP2, heparin cofactor 2 protein that is a thrombin inhibitor, and (6) C1QB, complement C1q subcomponent subunit that is one of the first components of the serum complement system. To determine the difference between the stress of the conditions, we compared the POST conditions.

To assess differences between post-stress plasma proteomes, we compared EANPOST to O_2_POST. For unfiltered reads, six proteins were significantly highly expressed in EANPOST, while six were significantly highly expressed in O_2_POST (*p* < 0.05). Proteins are listed in Table 8 and network analysis was completed for each set. For the six proteins highly expressed in EANPOST (vs. O_2_POST), five nodes were generated, collapsing splice isoforms or post-translational modifications. Significant interactions among the nodes were present (four edges, average local clustering coefficient 0.6, PPI enrichment *p* = 6.02 × 10^−5^). The only significant (FDR 0.0043) molecular function GO term was serine-type endopeptidase inhibitor activity (GO:0004867) and the one significantly enriched reactome pathway (FDR 0.0339) was an intrinsic pathway of fibrin clot formation. 

Among the six proteins more highly expressed in O_2_POST (vs. EANPOST), only two were identified as annotated species in reference databases. No network interactions were identified (two nodes, PPI enrichment *p*-value = 1.0). No functional enrichments were identified. However, one of these markers was apolipoprotein D (APOD) which is particularly recognized to be associated with regulating protection from oxidative stress [9,10,11].

## 4. Discussion

This pilot study of plasma proteome responses to prolonged diving in a dry hyperbaric environment breathing nitrox or hyperbaric oxygen revealed potential future directions for biomarker research for decompression and pulmonary oxygen toxicity stress. The main finding of this study is that 6.5 h breathing a nitrox mixture containing 30.6% O_2_ and 69.4% N_2_ at 2 ATA (10 msw) resulted in the presence of proteins that may be associated with hemostasis and coagulation pathways, while 6.5 h of hyperbaric oxygen exposure (100% O_2_) at 2 ATA results in activation of general pathways associated with innate immunity and immune signaling. One potential future target of interest that differed between EANPOST and O_2_POST was APOD. Future studies should determine whether APOD is a specific target for biomarker validation to physiological and respiratory clinical responses to pulmonary oxygen toxicity. Future work should also explore correlational analyses between plasma proteomics and clinical symptoms in protocols inducing robust clinical symptomology data.

Our findings might also have relevance to lung injury mechanisms. The cellular response to EAN and hyperoxic O_2_ type exposures related to increased reactive oxygen species are well-characterized. Local responses to ROS-induced damage in the lungs induce cytokine and chemokine signaling [12] that contributes to pulmonary edema if the oxygen exposures are prolonged. Among the immune responses that occur during acute lung injury, are alterations in coagulation and fibrin-signaling pathways. Animal models have demonstrated that pulmonary fibrin deposition, platelet accumulation, and lung-specific coagulation factors are upregulated during acute lung injury [13]. Anticoagulant treatments including antithrombin and heparin have successfully reduced the severity of inflammation during acute lung injury [14]. Lung epithelial cell-specific coagulation signaling mediates multiple aspects of acute lung injury pathophysiology, including traditional fibrinolysis [15,16,17]. Our observations regarding plasma proteins related to coagulation pathways are not confirmatory that acute lung injury or tissue damage that triggers pulmonary coagulation and fibrin deposition pathways are systemic in EAN, but it does provide some evidence for future directions in plasma biomarkers related to known mechanisms of lung or epithelium injury immune response. 

Our findings could also aid in the understanding of the metabolomic responses to venous gas emboli. During the EAN dive the body will uptake nitrogen leading to supersaturation of the tissues which will be subsequently off-gassed during decompression with the potential generation of venous gas emboli (VGE). VGE can induce a variety of biological effects through their interactions with blood [18] and has been associated with complement activation and decompression sickness susceptibility [19,20,21]. Although we did not verify that VGE were present following the EAN dive, our dive profile using the 30.6% O_2_ EAN breathing mixture was close to the edge of the no-decompression limit and it is therefore possible that VGE were generated in some of our subjects. Thus, the observation of plasma proteins related to coagulation pathways in EAN may be a response to microparticle generation during the dive [22] and/or VGE following decompression from nitrox gas [23] and not necessarily related to any lung hyperoxic stress from the EAN gas mixture. The lack of such response in O_2_POST is likely due to fact that during the HBO exposure the body will slowly denitrogenate without generating VGE and then on gas nitrogen following decompression to the surface.

The singular protein uniquely expressed in O_2_ vs. EAN at POST, APOD, is one of many apolipoproteins secreted and expressed in many tissues including respiratory epithelial cells (i.e., bronchus); it is a multi-ligand protein that supports many functions including those in oxidative stress and inflammation [24]. APOD has been well-studied [25] as an antioxidant with known structural mechanisms. The fact that APOD was uniquely expressed O_2_POST vs. EAN may reflect a difference in the nature of stress experienced in each condition. In a recently published study that investigated changes in the exhaled condensate of these same subjects, it was found that many of the compounds evident following the HBO dive were lipids or lipid-like molecules that are found in cell membranes and commonly serve to act as a membrane stabilizer [26]. The authors speculate that “the increased presence of many of these lipid-like molecules in the EBC of symptomatic subjects following the HBO dive may have resulted from tissue/cell damage in the lungs caused by the oxidative stress from reactive oxygen species overcoming antioxidative capacity leading to lipid peroxidation and the release of these compounds”.

The oxygen dose used in the current study during the O_2_ dive was greater than typically used during conventional hyperbaric oxygen therapy in order to induce noticeable stress on the pulmonary system. Our results show that there are wide inter-individual differences in the pulmonary stress responses to hyperbaric oxygen exposure that range from severe symptoms of pulmonary oxygen toxicity early on into the O_2_ dive to no symptoms at all during or after the O_2_ dive. It is unclear from our results if there is a direct link between pulmonary O_2_ toxicity symptomology and lung injury, as the oxygen exposures were only sufficient to induce the initial stages of pulmonary oxygen toxicity (i.e., tracheobronchitis) in approximately 71% of our diver subject population.

A medical follow-up on the day after the dive indicated that the pulmonary O_2_ toxicity symptoms had quickly resolved in most subjects. Four subjects did however indicate that they still had some mild irritation or “dryness” in their chest or upper airway that may have been associated with an inflammatory reaction of the pulmonary system to the hyperoxic stress from the dive. The timing of the post-dive blood draws in the current study was designed to ascertain if there are any early/acute biomarkers that would be associated with prolonged exposure to the nitrox or hyperbaric oxygen breathing mixtures. An analysis of the pulmonary function responses of the individual who experienced severe pulmonary oxygen toxicity symptoms following the O_2_ dive [27] suggests that the early onset of symptoms in this subject were likely neuronal in nature. As suggested by previous researchers, oxygen poisoning responses reflect a composite of both direct (i.e., neural effects) and indirect effects (i.e., inflammation, etc.) that will depend on the oxygen dosage received and the susceptibility of the organ system [28,29].

As the subject population in the current study were all males, we caution that susceptibility to pulmonary PO2tox and the resulting proteomic response to the different gas mixtures may be different in females than in males since there is some evidence in the literature that sex-specific hormones may play a role in modulating susceptibility to prolonged oxidative stress in males and females [30,31,32,33,34]. 

## 5. Conclusions

Our EAN data suggest that inflammatory/immune responses and coagulation pathways are prominent features of a systemic injury response following a prolonged nitrox dive and that future treatments or diving strategies that target these pathways may be promising directions for reducing hyperbaric decompression stress. 

While this study involved oxygen exposures that induced symptoms reflecting the early stages of pulmonary oxygen toxicity in some of our subjects (i.e., tracheobronchitis), the HBO exposure was not designed to induce lung injury that can occur with more prolonged oxygen exposures. However, it is possible that the proteomic responses found in our O_2_POST dive blood samples while reflecting an early antioxidant response may have been too early to detect an inflammatory reaction of the pulmonary tissues in the systemic circulation that may be evident one to two days following the hyperbaric oxygen exposure. Thus, future research should examine the proteome responses one to three days after the dive during the recovery phase to better define the metabolic profile and pathways that are ultimately affected by oxygen exposure. It will be critical in future work to not only characterize the clinical symptomology but also to appropriately measure stress-specific outcome variables defining the magnitude and type of oxidative stress and antioxidant response [35]. Additionally, studies permitting correlational or predictive analyses of proteomics results to clinical symptomology will support the translational aims of our -omics work.

**Table 1:** Significantly expressed proteins (*p* < 0.05) when comparing EANPRE and O2PRE and related publications in hyperbaria, hyperoxygen, or pulmonary oxygen toxicity for relevant protein results. Significantly expressed proteins that are associated with published literature relevant to hyperbaria, hyperoxygen, or pulmonary oxygen toxicity are marked with a, b, and/or c, respectively.

Accession (_HUMAN)	Ensembl ID (ENSG-)	Protein Name	Function	Relevant to Hyperbaria ^a^, Hyperoxygen ^b^, or Pulmonary O_2_ Toxicity ^c^?
EANPRE				
O2PRE				
FBLN1	00000077942	Fibulin-1	cell adhesion and migration along protein fibers within the extracellular matrix—haemostasis and thrombosis—owing to its ability to bind fibrinogen and incorporate into clots	-
TSP1	00000137801	Thrombospondin-1	adhesive glycoprotein mediating cell-to-cell and cell-to-matrix interactions, binds heparin, plays a role in ER stress response, via its interaction with the activating transcription factor 6 alpha (ATF6) producing adaptive ER stress response factors	a [36,37]
KVD11	00000211632	Immunoglobulin kappa variable 3D-11	V region of the variable domain of immunoglobulin light chains that participates in the antigen recognition	-
PLF4	00000163737	Platelet factor 4	released during platelet aggregation, neutralizes the anticoagulant effect of heparin, chemotactic for neutrophils and monocytes, and inhibits endothelial cell proliferation	-
CXCL7	00000163736	Platelet basic protein	stimulates DNA synthesis, mitosis, glycolysis, intracellular cAMP accumulation, prostaglandin E2 secretion, and synthesis of hyaluronic acid, and sulfated glycosaminoglycan	-

**Table 2:** Gene ontology terms significantly associated to molecular functions and biological processes for differential protein results from EANPRE and O2PRE.

ID	Term Description	FDR	Matching Proteins in Your Network (Labels)
GO:0070051	Fibrinogen binding	0.0015	THBS1,FBLN1
GO:0045236	CXCR chemokine receptor binding	0.0096	PPBP,PF4
GO:0001968	Fibronectin binding	0.0137	THBS1,FBLN1
GO:0002576	Platelet degranulation	0.0153	THBS1,PPBP,PF4
GO:0005102	Signaling receptor binding	0.031	THBS1,PPBP,PF4,FBLN1
GO:0008009	Chemokine activity	0.031	PPBP,PF4

**Table 3:** Gene ontology terms significantly associated to reactome pathways for differential protein results from EANPRE and O2PRE.

ID	Term Description	FDR	Matching Proteins in Your Network (Labels)
HSA-114608	Platelet degranulation	0.0025	THBS1,PPBP,PF4
HSA-380108	Chemokine receptors bind chemokines	0.029	PPBP,PF4
HSA-8936459	RUNX1 regulates genes involved in megakaryocyte differentiation and platelet function	0.0309	THBS1,PF4

**Table 4:** Significantly expressed proteins (*p* < 0.05) when comparing O2PRE and O2POST and related publications in hyperbaria, hyperoxygen, or pulmonary oxygen toxicity for relevant protein results. Significantly expressed proteins that are associated with published literature relevant to hyperbaria, hyperoxygen, or pulmonary oxygen toxicity are marked with a, b, and/or c, respectively.

Accession (_HUMAN)	Ensembl ID (ENSG-)	Protein Name	Function	Relevant to Hyperbaria ^a^, Hyperoxygen ^b^, or Pulmonary O_2_ Toxicity ^c^?
O2PRE				
A0A087WTE4	00000149294	Neural cell adhesion molecule 1	a cell adhesion molecule involved in neuron–neuron adhesion, neurite fasciculation, outgrowth of neurites, etc.	-
CATA	00000121691	Catalase	serves to protect cells from the toxic effects of hydrogen peroxide, promotes growth of cells including T-cells, B-cells, myeloid leukemia cells, melanoma cells, and mastocytoma cells	a, b, c [38,39,40,41,42,43,44]
HBB	00000244734	Hemoglobin subunit beta	involved in oxygen transport from the lung to the various peripheral tissues	b, c [45,46,47]
FBLN1	00000077942	Fibulin-1	cell adhesion and migration along protein fibers within the extracellular matrix. haemostasis and thrombosis—owing to its ability to bind fibrinogen and incorporate into clots	-
C1QB	00000173369	Complement C1q subcomponent subunit B	C1q associates with the proenzymes C1r and C1s to yield C1, the first component of the serum complement system	-
CO8B	00000021852	Complement component C8 beta chain	constituent of the membrane attack complex that plays a key role in the innate and adaptive immune response by forming pores in the plasma membrane of target cells	-
F5GY80	00000021852	Complement component C8 beta chain	constituent of the membrane attack complex that plays a key role in the innate and adaptive immune response by forming pores in the plasma membrane of target cells	-
C9FPQ9	00000171557	Fibrinogen gamma chain	binds through its gamma chains to cell surface receptors, growth factors, and coagulation factors to perform its key roles in fibrin clot formation, platelet aggregation, and wound healing	-
A0A0C4DGZ8	00000185245	Glycoprotein Ib (Platelet), alpha polypeptide	a receptor for von Willebrand disease (VWF)	-
O2POST				
APOD	00000189058	Apolipoprotein D	occurs in the macromolecular complex with lecithin-cholesterol acyltransferase, most likely involved in the transport and binding of bilin, able to transport a variety of ligands in several different contexts	c [14,48]

**Table 5:** Gene ontology terms significantly associated to biological processes for the 9 highly expressed proteins (*p* < 0.05) results in O2PRE (vs. O2POST).

ID	Term Description	FDR	Matching Proteins in Your Network (Labels)
GO:0072378	Blood coagulation, fibrin clot formation	0.0021	GP1BA,FBLN1,FGG
GO:0070527	Platelet aggregation	0.0027	GP1BA,HBB,FGG
GO:0006950	Response to stress	0.0033	CAT,C1QB,GP1BA,FBLN1,HBB,FGG,C8B,NCAM1
GO:0007596	Blood coagulation	0.0085	GP1BA,FBLN1,HBB,FGG
GO:0042730	Fibrinolysis	0.036	GP1BA,FGG

**Table 6:** Significantly expressed proteins (*p* < 0.05) when comparing EANPRE and EANPOST and related publications in hyperbaria, hyperoxygen, or pulmonary oxygen toxicity for relevant protein results. Significantly expressed proteins that are associated with published literature relevant to hyperbaria, hyperoxygen, or pulmonary oxygen toxicity are marked with a, b, and/or c, respectively.

Accession (_HUMAN)	Ensembl ID (ENSG-)	Protein Name	Function	Relevant to Hyperbaria ^a^, Hyperoxygen ^b^, or Pulmonary O_2_ Toxicity ^c^?
EANPRE				
CON__ENSEMBL:ENSBTAP00000024146	unclassified	CON__ENSEMBL:ENSBTAP00000024146	unknown, not in the protein database	-
KV105	00000243466	Immunoglobulin kappa variable 1–5	V region of the variable domain of immunoglobulin light chains that participates in the antigen recognition	-
HBD	00000223609	Hemoglobin subunit delta	involved in oxygen transport from the lung to the various peripheral tissues	-
PZP	00000126838	Pregnancy zone protein	contains a ‘bait region’ which has specific cleavage sites for different proteinases that is able inhibit all four classes of proteinases by unique ‘trapping’ mechanism	-
EANPOST				
A0A087WTE4	00000149294	Neural cell adhesion molecule 1	a cell adhesion molecule involved in neuron–neuron adhesion, neurite fasciculation, outgrowth of neurites, etc.	-
CON_Q6T181	unclassified	Sex hormone-binding globulin	contaminant protein from bovine	-
APOH	00000091583	Beta-2-glycoprotein 1	also known as apolipoprotein H, binds to various kinds of negatively charged substances such as heparin, phospholipids, and dextran sulfate; may prevent activation of the intrinsic blood coagulation cascade by binding to phospholipids on the surface of damaged cells	-
G3XAK1	00000173531	Hepatocyte growth factor-like protein	has a major role in embryonic organ development, specifically in myogenesis, in adult organ regeneration, and in wound healing	-
AMBP	00000106927	Protein AMBP	inter-alpha-trypsin inhibitor inhibits trypsin, plasmin, and lysosomal granulocytic elastase; inhibits calcium oxalate crystallization	-
FA9	00000101981	Coagulation factor IX	a vitamin K-dependent plasma protein that participates in the intrinsic pathway of blood coagulation by converting factor X to its active form in the presence of Ca^2+^ ions, phospholipids, and factor 8a	-
FA11	00000088926	Coagulation factor XI	triggers the middle phase of the intrinsic pathway of blood coagulation by activating coagulation factor IX	-
HEP2	00000099937	Heparin cofactor 2	predominant thrombin inhibitor in place of antithrombin III, also inhibits chymotrypsin	-
C9JV77	00000145192	Alpha-2-HS-glycoprotein	promotes endocytosis, possesses opsonic properties, and influences the mineral phase of bone	-
TTHY	00000118271	Transthyretin	thyroid hormone-binding protein, transports thyroxine from the bloodstream to the brain	a [49,50]
HRG	00000113905	Histidine-rich glycoprotein	plasma glycoprotein that binds a number of ligands such as heparin, heparan sulfate, thrombospondin, plasminogen, and divalent metal ions	-
S10A9	00000163220	Protein S100-A9	a calcium- and zinc-binding protein which plays a prominent role in the regulation of inflammatory processes and immune response; has oxidant-scavenging activities	-

**Table 7:** Gene ontology terms significantly associated with molecular functions and biological processes 12 highly expressed proteins (*p* < 0.05) results in EANPOST (vs. EANPRE).

ID	Term Description	FDR	Matching Proteins in Your Network (Labels)
GO:0007597	Blood coagulation, intrinsic pathway	0.0011	APOH,F9,F11
GO:0051917	Regulation of fibrinolysis	0.0011	APOH,HRG,F11
GO:0007596	Blood coagulation	0.0017	APOH,SERPIND1,F9,HRG,F11
GO:0019538	Protein metabolic process	0.0031	APOH,SERPIND1,F9,TTR,AMBP,S100A9,F11,AHSG,MST1,NCAM1
GO:0030195	Negative regulation of blood coagulation	0.0031	APOH,HRG,F11
GO:0031638	Zymogen activation	0.0031	APOH,F9,F11
GO:0052548	Regulation of endopeptidase activity	0.0031	SERPIND1,HRG,AMBP,S100A9,AHSG
GO:0032101	Regulation of response to external stimulus	0.0052	APOH,HRG,S100A9,F11,AHSG,MST1
GO:0016192	Vesicle-mediated transport	0.0073	APOH,F9,HRG,TTR,AMBP,S100A9,AHSG
GO:0051918	Negative regulation of fibrinolysis	0.0087	APOH,HRG
GO:0031639	Plasminogen activation	0.0099	APOH,F11
GO:0045055	Regulated exocytosis	0.0099	APOH,HRG,TTR,S100A9,AHSG
GO:0051336	Regulation of hydrolase activity	0.0116	APOH,SERPIND1,HRG,AMBP,S100A9,AHSG
GO:0002576	Platelet degranulation	0.0174	APOH,HRG,AHSG
GO:0080134	Regulation of response to stress	0.0189	APOH,HRG,AMBP,S100A9,F11,AHSG
GO:0050790	Regulation of catalytic activity	0.0274	APOH,SERPIND1,HRG,AMBP,S100A9,AHSG,MST1
GO:0006950	Response to stress	0.0308	APOH,SERPIND1,F9,HRG,S100A9,F11,AHSG,NCAM1
GO:0006935	Chemotaxis	0.0452	SERPIND1,HRG,S100A9,NCAM1
GO:0004866	Endopeptidase inhibitor activity	0.0064	SERPIND1,HRG,AMBP,AHSG
GO:0008201	Heparin binding	0.0064	APOH,SERPIND1,HRG,F11
GO:0004867	Serine-type endopeptidase inhibitor activity	0.0086	SERPIND1,HRG,AMBP
GO:0004252	Serine-type endopeptidase activity	0.0258	F9,F11,MST1
GO:0019865	Immunoglobulin binding	0.0258	HRG,AMBP
GO:0030234	Enzyme regulator activity	0.0356	APOH,SERPIND1,HRG,AMBP,AHSG

**Table 8:** Significantly expressed proteins (*p* < 0.05) when comparing EANPOST and O2POST and related publications in hyperbaria, hyperoxygen, or pulmonary oxygen toxicity for relevant protein results. Significantly expressed proteins that are associated with published literature relevant to hyperbaria, hyperoxygen, or pulmonary oxygen toxicity are marked with a, b, and/or c, respectively.

Accession (_HUMAN)	Ensembl ID (ENSG-)	Protein Name	Function	Relevant to Hyperbaria ^a^, Hyperoxygen ^b^, or Pulmonary O_2_ Toxicity ^c^?
EANPOST				
A0A087WTE4	00000149294	Neural cell adhesion molecule 1	a cell adhesion molecule involved in neuron–neuron adhesion, neurite fasciculation, outgrowth of neurites, etc.	-
AMBP	00000106927	Protein AMBP	inter-alpha-trypsin inhibitor inhibits trypsin, plasmin, and lysosomal granulocytic elastase; inhibits calcium oxalate crystallization	-
CON__ENSEMBL:ENSBTAP00000031900	unclassified	CON__ENSEMBL:ENSBTAP00000031900	unknown, not in the protein database	-
FA9	00000101981	Coagulation factor IX	a vitamin K-dependent plasma protein that participates in the intrinsic pathway of blood coagulation by converting factor X to its active form in the presence of Ca^2+^ ions, phospholipids, and factor 8a	-
HEP2	00000099937	Heparin cofactor 2	predominant thrombin inhibitor in place of antithrombin III, also inhibits chymotrypsin	-
ITIH4	00000055955	Inter-alpha-trypsin inhibitor heavy chain H4	involved in inflammatory responses to trauma, may also play a role in liver development or regeneration	-
O2POST				
A0A0A0MS08	00000211896	Immunoglobulin heavy constant gamma 1	constant region of immunoglobulin heavy chains	-
A0A286YFJ8	00000211892	Immunoglobulin heavy constant gamma 4	constant region of immunoglobulin heavy chains	-
APOD	00000189058	Apolipoprotein D	occurs in the macromolecular complex with lecithin-cholesterol acyltransferase, most likely involved in the transport and binding of bilin, able to transport a variety of ligands in several different contexts	c [48]
KV105	00000243466	Immunoglobulin kappa variable 1–5	V region of the variable domain of immunoglobulin light chains that participates in the antigen recognition	-
KV315	00000244437	Immunoglobulin kappa variable 3–15	V region of the variable domain of immunoglobulin light chains that participates in the antigen recognition	-
M0R2W8	00000161031	N-acetylmuramoyl-L-alanine amidase (Fragment)	an enzyme that catalyzes a chemical reaction that cleaves the link between N-acetylmuramoyl residues and L-amino acid residues in certain cell-wall glycopeptides	-

## Data Availability

Not applicable.
